# Torque Teno Virus: Lights and Shades

**DOI:** 10.3390/v17030334

**Published:** 2025-02-27

**Authors:** Paola Brani, Hafza Zahira Manzoor, Pietro Giorgio Spezia, Andrea Vigezzi, Giuseppe Ietto, Daniela Dalla Gasperina, Claudia Minosse, Annalisa Bosi, Cristina Giaroni, Giulio Carcano, Fabrizio Maggi, Andreina Baj

**Affiliations:** 1 Department of Medicine and Technological Innovation, University of Insubria, 21100 Varese, Italy; 2 Laboratory of Microbiology, ASST Sette Laghi, 21100 Varese, Italy; 3 Laboratory of Virology and Biosafety Laboratories, National Institute for Infectious Diseases “Lazzaro Spallanzani”-IRCCS, 00149 Rome, Italy

**Keywords:** anelloviruses, torque teno virus (TTV), viral load, solid organ transplant, immunocompromised, immune marker, transplantation

## Abstract

Torque Teno Virus (TTV) is a highly prevalent non-pathogenic DNA virus whose plasma levels may be related to the host’s immune status. TTV gained attention about 25 years ago, but its replication is not fully understood, nor is its relationship with the host's immune system. Despite this lack of knowledge, TTV is currently being investigated as a functional biomarker of the immune system in patients with immunological damage and inflammatory diseases. Monitoring TTV viral load over time may help clinicians in making therapeutic decisions regarding immunosuppression as well as the likelihood of infectious complications. This review summarizes what we do and do not know about this enigmatic virus.

## 1. Introduction

It was in 1997 when a paper entitled “A novel DNA virus (TTV) associated with elevated transaminase levels in post-transfusion hepatitis of unknown etiology” was published by Nishizawa and coworkers [1]. First identified as a new hepatitis virus, TT virus (TTV) was named after the initials of a Japanese patient with unexplained post-transfusion hepatitis. In 2004, the meaning of TTV was revised to stand for Torque Teno Virus, derived from the Latin words “torques” and “tenuis,” meaning necklace and thin, respectively, in reference to the structure of its genome. This renaming followed the International Committee on Taxonomy of Viruses (ICTV) rule prohibiting official virus names from being based on a person’s name [2]. As medical history has taught us that viruses have always been associated with the development of disease, researchers scrambled to assign an illness to this new virus. But, TTV seemed to be a mysterious virus, orphaned by disease.

With the advent of DNA sequencing showcasing a significant amount of viral “dark matter”, TTV emerged from being a novel hepatitis virus to an orphan disease agent, ultimately becoming a key element of the human virome [3]. Since the publication of the first paper in 2002 [4], the field of virome research has significantly advanced. As researchers define the virome as the viral component of the human microbiome, the extensive realm of the human virome is gradually being deciphered, including our understanding of TTV.

The current picture is that TTV is a pantropic and ubiquitous virus present in up to 98% of the population [5]. Importantly, the emphasis lies not on positivity but on the concentration of the virus. Plasma viral loads span a range that is considered “normal”. Deviations from this range, whether increased or decreased, signal a pathological condition in which TTV is not the causative agent but acts as an indicator of immune system performance.

Since TTV was first identified in 1997, many similar viruses have been found and classified within the family *Anelloviridae*, which are found in most healthy humans. Of particular interest is the fact that co-infections with multiple unique lineages are also common, which together constitute a “personal anellome”, a kind of fingerprint [6,7,8,9]. The full extent of human anellovirus (AV) diversity, the “global anellome”, and the mechanism(s) contributing to its diversification remain largely uncharacterized [10,11]. 

TTV has evolved from a novel hepatitis virus to an orphan pathogen and, ultimately, to a key element of the human virome [6].

Present in the plasma of most of the world’s population, TTV is now considered a long-lived human passenger. However, there are still many unanswered questions. 

## 2. A Step Back: From the Clinic to Basic Virology

**Figure 1:**
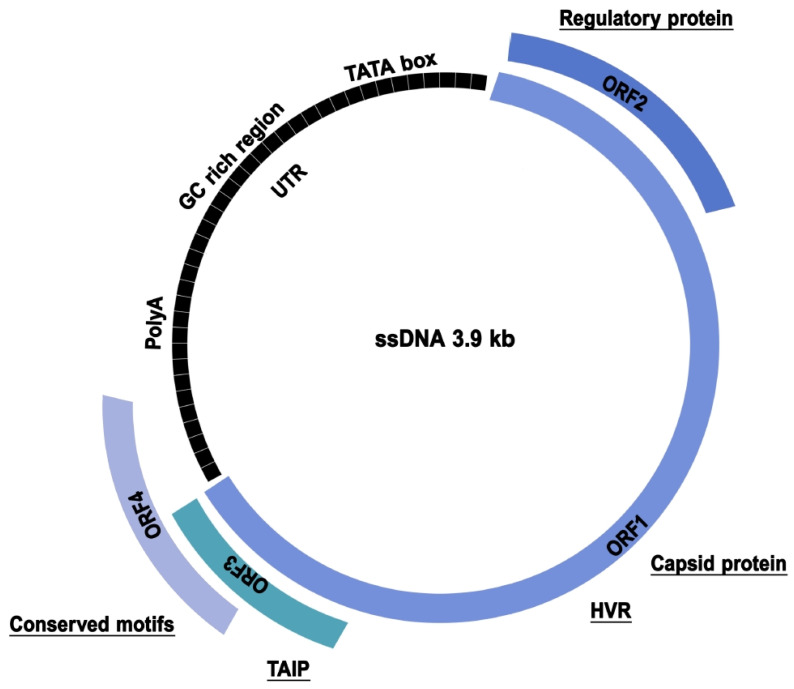
TTV genome organization. The conserved non-coding region (UTR) and the coding region sequence are illustrated. ORF1 is responsible for the transcription of the capsid protein and contains hypervariable regions (HVRs). ORF2 exerts regulatory effects on both innate and adaptive immunity, as well as the inflammatory response. ORF3 is responsible for the transcription of the TTV-derived apoptosis-inducing protein (TAIP), while ORF4 is a protein whose function remains to be elucidated.

TTV has been described in the published literature as a small, non-enveloped, and icosahedral virus with a diameter of 30 to 32 nm and a single-stranded, negative-sense, and circular DNA genome of 3.5 to 3.9 kilobases (kb). Its replication occurs via a rolling-circle mechanism, common to other circular DNA viruses, and employs host polymerases [12]. Further examination of the viral genome reveals a highly conserved non-coding region (UTR) of 1.2 kb, which is responsible for replication and expression control, while the remaining 2.6 kb represents the coding region sequence (CDS) [12,13]. In addition, at least four partially overlapping open reading frames (ORFs) have been identified [14,15,16], along with small ORFs of unknown function, including a hypervariable region (HVR) and an N-terminal poly-A-arginine fragment sequence [17]. ORF1 (700 aa) encodes the capsid protein and is thought to be involved in TTV spin-circle replication, and its core domain appears to be a mediator of binding to genomic DNA and transport to the nucleus [18]. In contrast to the other proteins, ORF1 possesses hypervariable regions (HVRs), where mutations leading to amino acid changes occur more frequently, suggesting the potential for immune evasion by hypermutation [19,20]. Zheng et al. investigated the function of ORF2 (200 aa), revealing its role in suppressing the NF-κB pathway, thereby exerting regulatory effects on both innate and adaptive immunity, as well as the inflammatory response [15]. ORF3, which is conserved within a limited group of the heterogeneous TTV population, encodes a protein called TTV-derived apoptosis-inducing protein (TAIP), which is known to be able to induce p53-independent apoptosis in various human hepatocellular carcinoma cell lines [21]. Sequence analysis predicts the presence of additional potential ORFs that may encode poorly known proteins (Figure 1). In human TTV, a fourth ORF (ORF4) has been observed to encode 70 amino acids with a conserved motif (EX8RX2RX6PX12-19FX1L), though its function remains to be elucidated [13].

It is evident that the comprehension of the TTV genome has been and continues to be of fundamental importance, given that most studies on TTV are primarily based on the research and quantification of the viral genome in different samples using molecular methods.

In 1999, it was hypothesized that TTV replicates in the liver, given the higher concentration of TTV DNA found in bile compared to peripheral blood [18]. Concurrently, it was proposed that TTV can infect and replicate in bone marrow cells, as the amount of TTV DNA in serum samples decreases to the point of being undetectable during the myelosuppression period following bone marrow transplantation in most TTV-infected patients [22,23]. Subsequent studies concentrated on the tropism of TTV for T and B lymphocytes, monocytes, and natural killer lymphocytes, as well as granulocytes and other polymorphonuclear cells [24,25]. 

Indeed, TTV genomes have been identified in a cohort of blood transfusion patients who were subject to close monitoring, as well as in metagenomic datasets from a variety of other sample types, including stool [26], urine [27], bone marrow [28], liver [29,30], lungs [6], lymphoid tissue [6], and oropharyngeal and/or salivary glands [31].

This prompts the question of whether TTV has a broad or specific host range. Given its presence in various samples, it might either recognize a common receptor and be pantropic or primarily infect leukocytes, leading to its widespread distribution [32]. Data obtained by Kosulin et al. [33] demonstrate that virus replication occurs in CD15+ cells, the most abundant fraction of granulocytes, suggesting that sufficiently high granulocyte numbers may be a prerequisite for efficient TTV expansion. The same study demonstrated a significant correlation between TTV copy numbers and neutrophil count. Conversely, the effect of anti-T lymphocyte drugs on TTV levels provided the basis for the suggestion that T lymphocytes are the host cells for TTV replication [34].

Interestingly, many studies have shown that certain TTV types may persist in PBMC after serum clearance [35,36], suggesting a type-dependent infection of PBMC. It is plausible that certain TTV types may have a predilection for hematopoietic cells, while others have an affinity for hepatocytes [37]. It is noteworthy that, in healthy individuals, TTV is predominantly present in granulocytes compared to other peripheral blood cell types [38].

Also, the high prevalence and the viral load of the virus in saliva suggested that it could also replicate in salivary glands and/or oropharyngeal tissues [31]; on the contrary, the almost-leak of virus load in cerebrospinal fluid hints that the central nervous system is less likely to be the site of viral replication [11,38].

In the search for a carrier of TTV, several options should be considered: T cells cannot be excluded as a site of persistence [34], but another option has been considered as TTV has been identified in plasma-depleted platelet concentrates, raising the possibility that TTV may be taken up by or adhered to platelets [33]. 

However, there is another factor to consider: viral persistence necessitates infecting long-lived cells, whereas viral replication, typically linked with cell destruction, can take place in short-lived cells that offer a suitable setting for the virus to reproduce.

For this reason, it is possible that peripheral granulocytes, although an important site of TTV replication, may not serve as host cells for the persistence of the virus because of their very short life span. 

Until a cellular model for the persistence and replication of TTV in vitro can be established, its interaction with the human host will remain in the shadows. 

Soon after its discovery, several attempts were made to isolate TTV by replication in cell culture. On the other hand, this has always been the classical virological approach to learning the molecular details of viral replication and interaction with the host. 

It is rather unexpected that TTV still resists all efforts for in vitro replication and isolation. This situation could be considered paradoxical, considering the extensive clinical research on the virus and the relatively scant basic knowledge we have obtained about it. Surprisingly, TTV continues to elude all attempts at in vitro replication and isolation. 

In 2001, Maggi and coworkers demonstrated reproductive infection in stimulated PBMC cultures [4,33]. The same observation was made in 2005 in PHA-stimulated PBMC cultures and the Raji lymphoblast cell line [29]. The same authors also showed that the Chang liver cell line, derived from non-malignant liver tissue, supports the growth of TTV, also showing a marked cytopathic effect, but with low levels of viral particles secreted in the supernatant fluid. On the contrary, the virus failed to establish a productive infection in the two HCC cell lines HepG2 and MH1C1.

Other attempts to replicate AVs in vitro have been unsuccessful. Human cell lines, such as the Chang liver and Raji cell lines [36], along with HEK293TT, lymphoma, and T-cell leukemia, have shown evidence of TTV infection in initial passages. However, the virus failed to replicate in subsequent passages [26,39,40].

One constant in vitro replication attempt of TTV is the lack of a true viral titer elevation indicative of its replication and cellular release. The other feature is the failure of any attempt to establish persistent infection. Unlike in vivo infection, in vitro infection by TTV is lost approximately two weeks after infection.

This inability to establish a persistent viral infection makes it extremely difficult to discover its receptor and, thus, its tropism, as well as to understand the intracellular factors that modulate its replication and, ultimately, the nature of the close relationship between TTV and the immune system.

## 3. The Origins of TTV: Where Does It Come from?

Following the discovery of TTV, numerous types of AV have been identified [12,41,42] in both humans and various mammals [14,43,44,45,46,47,48]. As of September 2023, the International Committee on Taxonomy of Viruses (ICTV) ratified 173 species classified in 34 genera and, in the same year, Butkovic and colleagues [49] proposed the placement of AVs in a new phylum, designated “Commensaviricota”, within the kingdom Shotokuvirae.

The same authors studied the evolution of AVs through a comparative structural analysis of their signature ORF1 proteins, observing considerable variability in the overall size of this protein among different AV genera. Notably, a systematic comparison of their structures suggests that the AV ORF1 protein has evolved from an ancestral virus through incremental increases in size and structural complexity. Profile–profile comparison has shown that AV ORF1 proteins are homologous to the capsid proteins of circoviruses, the only other small, circular, and ssDNA viruses known to infect mammals [50], suggesting that AVs evolved from a circovirus-like ancestor. It is still not clear whether this virus was a member of the *Circoviridae* family or whether another virus was the ancestor of both circoviruses and AVs.

**Figure 2:**
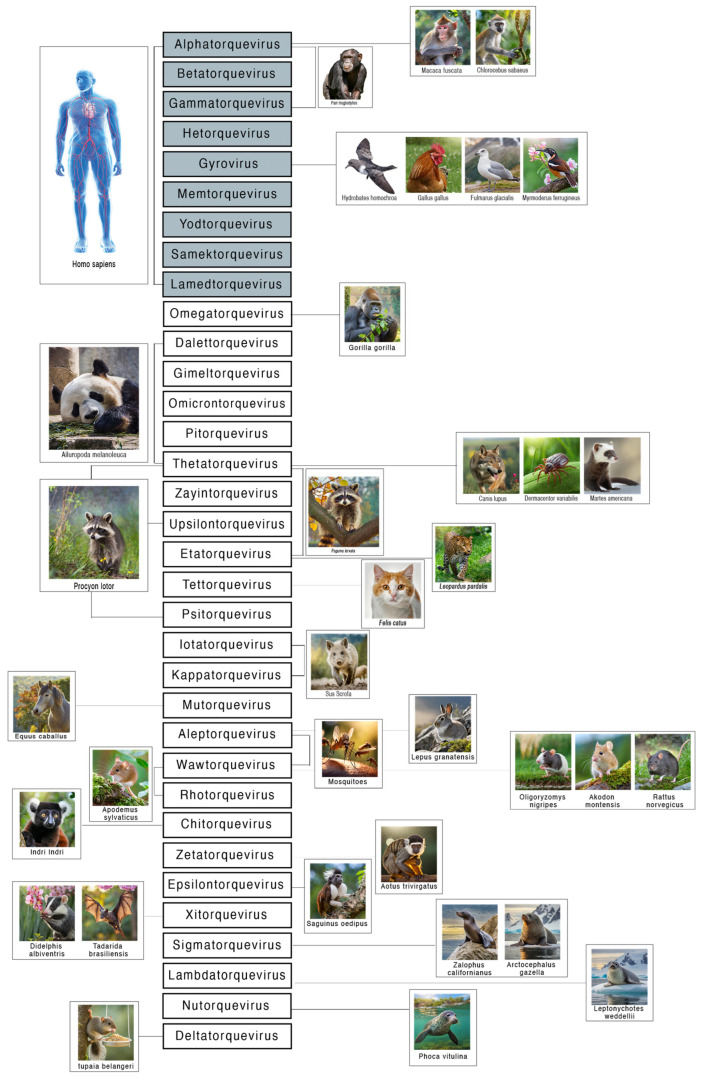
*Anelloviridae* genera and their hosts. The 34 genera of the family *Anelloviridae* are shown. The nine genera identified as capable of infecting humans are shown in grey: Alphatorquevirus, Betatorquevirus, Gammatorquevirus, Hetorquevirus, Gyrovirus, Lamedtorquevirus, Memtorquevirus, Samektorquevirus, and Yodtorquevirus. For the other genera, mammalian and invertebrate hosts are indicated. As shown, some genera have a preferred host, while others have an extended host tropism.

Previously classified in the family *Circoviridae* [14,43], the novel family *Anelloviridae* was introduced in 2009. This classification was derived from the Latin word “anellus”, meaning “ring”, and was chosen to signify the circular genome of all members of the family. Although the taxonomy of AVs is constantly being revised, it appears that only nine genera have been identified as capable of infecting humans: Alphatorquevirus (TTV), Betatorquevirus (TT mini viruses—TTMV), Gammatorquevirus (TT midi viruses—TTMDV), Hetorquevirus [43], Gyrovirus, and, more recently, Lamedtorquevirus, Memtorquevirus, Samektorquevirus, and Yodtorquevirus [45] (Figure 2). The prevailing view is that alpha, beta, and gamma viruses are commensal components of the human virome [7] and are predominantly acquired during early childhood [51]. In addition, research has shown that TTV genomic diversity increases with age [52], but it is unclear whether this pattern persists into adulthood or how long it lasts. Co-infection with different AV species and genotypes within the same genus is also possible [27]. The discovery of viral diversity and the identification and characterization of non-human AVs, which are particularly important for understanding viral evolutionary dynamics, have been profoundly impacted by the advent of high-throughput sequencing (HTS) technologies and metagenomic protocols. 

Currently, AVs have been identified in several mammals (Hominidae, Canidae, Felidae, Suidae, Muridae, Ursidae, Molossidae, and Phocidae), but also in invertebrates such as Culicidae [46,47,48,53,54,55]. 

Interestingly, non-human AVs do not appear to be pathogenic, with the exception of Gyrovirus chicken anemia, which causes anemia, intramuscular hemorrhage, lymphoid atrophy, and bone marrow aplasia in chickens [56]. 

Characterizing of the diversity of AVs and their natural hosts is central to understanding their host range and the dynamics of virus transmission in nature. It has been hypothesized that AVs are mainly spread by fecal–oral transmission [57] and saliva [58,59], but it is possible that other ways of transmission, both intra- and inter-species, are possible. 

An analysis of the distribution of the different genera of AVs shows that some have a preferred host (such as Lambdatorquevirus, which, according to current knowledge, is only found in Phocidae and Otariidae), while others have a wide host tropism, such as Thetatorquevirus, which infects mammals such as Ursidae, but also Canidae, Mustelidae, and even Ixodidae. The latter, also known as the American dog tick or wood tick, is a species of tick known to transmit the bacteria responsible for several human diseases, including Rocky Mountain spotted fever and tularemia (*Francisella tularensis*). It is speculative whether the presence of anellovirus in the wood tick is the result of a blood meal from an individual infected with thetatorquevirus, e.g., Canis lupus [52]. 

## 4. TTV: Not a Causative Factor but a Marker

A PubMed search using the keywords (Anellovirus) OR (Torque Teno Virus) shows that 1338 articles have been published since the discovery of the virus in 1997. It is noteworthy that, from 1998 to 2003, and, to a lesser extent, until 2010, many articles focused on the potential role of TTV as an etiologic agent of liver disease. Subsequently, the focus gradually shifted to the concept of TTV as a “commensal virus”, defined as a component of the human virome that is not known to cause pathology in humans.

The earliest known discussion of the virome can be traced back to 2002. About a decade later, the first paper linking TTV to the virome was published. Since 2012, numerous papers have been published discussing the potential of TTV as a marker for the immunosuppression status in the post-transplant period.

It is now known that TTV is highly prevalent, accounting for 97% of all anelloviruses, and represents a significant proportion (68%) of the blood virome in many solid organ transplant recipients [27]. As its replication is under the control of a functioning immune system, it is hypothesized that the quantification of the viral load in the blood may serve as a potential indicator of its functionality [27]. Results to date suggest that, in the presence of a competent immune system, the circulating levels of TTV are approximately 10^2^–10^3^ copies/mL [60], which is considered to be within the normal range. Most studies investigating TTV have shown an association between unfavorable outcomes or disease progression and TTV loads that are either too low or too high [61,62,63].

Its presence has been extensively studied in transplant patients, with the aim of ensuring proper post-transplant management and avoiding the two most feared complications: infection and organ rejection [27]. A search using the keywords (Anellovirus) OR (Torque Teno Virus) AND (transplant) yielded 225 results, with a gradual increase from 2014 to the present. Most of these papers focus on the correct management of immunosuppression, which is important for long-term care, to prevent the development of both opportunistic infections [61] and cancer [64]. The correlation between TTV, induced immunosuppression, and the infection risks directly associated with this condition showed that individuals with severely compromised immune systems have a higher TTV load. In contrast, patients receiving inadequate doses of immunosuppressive drugs have a low TTV load [65]. Opportunistic infections, including Cytomegalovirus (CMV), BK virus (BKV), and Epstein–Barr virus (EBV), have the potential to cause virus-specific disease or even graft loss, or post-transplant lymphoproliferative disease [64]. Research has shown a significant association between BKV, CMV, and TTV. In particular, increased immunosuppression correlates with increased viral loads of both pathogenic viruses and TTV [61,63,66]. Some studies suggest that an elevated TTV plasma load may occur in the pre-infection period, suggesting its potential use as a predictive marker. However, other studies have not found a consistent relationship between TTV levels and viral infections [67,68]. The current focus is on determining the optimal TTV load for predicting infection in order to prevent adverse events [69].

Conversely, TTV appears to be a promising indicator for the detection of graft rejection. Studies have shown that TTV plasma levels are significantly lower in the presence of rejection at only 25% of the levels observed in the absence of rejection for both kidney and lung transplantation [70,71,72]. There is also a lack of agreement on this issue. Most studies suggest an inverse association between rejection and TTV burden; one found no association between TTV burden and rejection [66], and this study could not replicate its earlier findings. Some studies reported reduced odds or hazards associated with increased TTV burden [62,72,73,74,75,76], or found a lower TTV load in patients who experienced rejection compared to those who did not [76,77].

Given the complex interactions between the immune system and a variety of disease processes, the applicability of TTV may be extended to broader immunodeficiencies, inflammatory processes, and cancers [78,79,80].

Indeed, another factor that has been investigated over the years is the association between TTV and tumors. A PubMed search using the terms (Anellovirus) OR (Torque Teno Virus) AND (tumor) yielded 101 papers published from 1998 to the present. 

Some viruses are known to have oncogenic properties, and for many, the mechanisms by which they increase cell proliferation and/or inhibit apoptosis are known. EBV, HBV, HCV, HPV, HHV-8, and HTLV-1 and 2 are among the viruses associated with cancer pathology [81], in line with the 2.2 million cancer cases attributed to infection in 2018 [8]. In the first period after the discovery of TTV, in the years between 1998 and the early 2000s, scientific activity focused on the presence of TTV in the serum of patients with non-B, non-C hepatitis, and its possible role in non-B non-C hepatocellular carcinoma (HCC). Possible transmission by transfusion was emphasized, but it soon became apparent that TTV was present in different biological samples, suggesting different modes of transmission. The assessment of the presence of TTV over time also shows that the virus can cause persistent infection, strengthening the hypothesis of its possible oncogenic role [82]. 

Several studies have attempted to correlate TTV with various cancer pathologies, from hepatocarcinoma to HPV-related cancers, breast cancer, non-Hodgkin’s lymphoma, and colorectal cancer, but have yielded inconsistent results [83,84,85]. 

The turning point came in 2007, when scientists began to question whether TTV, which is widespread and found in over 90 percent of the world’s adult population without any known pathology, might be associated with various inflammatory conditions [86]. In 2007, Zheng H. and coworkers [15] correlated the activity of the ORF2 protein in the regulation of innate and adaptive immune response, but were still searching for a pathogenetic mechanism that could correlate TTV with a pathological state. 

In 2016, nine years later, Hettmann A. investigated the presence of TTV in saliva and biopsy samples from patients with head and neck cancer (HNCC), oral cancer, and controls. The study concluded that “our data are compatible with the idea that TTV may act as a cocarcinogen in certain cases of HNCC. Alternatively, HNCC may facilitate either TTV replication or TTV entry into saliva” [81]. 

This started an ongoing debate: is TTV a cause or just a sentinel? When TTV was classified as part of the virome [87], it went from being a potentially oncogenic virus to an enigmatic one, lacking pathology but possibly indicative of immune system function, even in cancer patients [78,88,89,90] (Table 1).

## 5. TTV: A Longtime Human Passenger

Human infections have been shown to occur at an early age [65] and have been identified in almost all human tissue [32,87], consistent with the lymphocytes being the primary site of AV replication [34]. It has been suggested that AVs positively influence human health by shaping immunity during early development [32], and are now considered part of the ‘healthy human virome’ [12,282], likely due to the extensive co-evolution of AVs with their mammalian hosts [283,284,285,286].

Numerous studies have suggested horizontal and vertical routes of TTV transmission. Horizontal transmission includes parenteral, fecal–oral, and sexual routes. Vertical transmission includes the possible passage of virus from mother to fetus during pregnancy and breastfeeding [287]. However, while the potential for vertical transmission has been suggested, alphatorquevirus DNA has not been consistently detected in umbilical cord blood. This finding supports the conclusion that transplacental transmission of AVs cannot be efficient [287]. It can be hypothesized that AVs may be transmitted by breastfeeding, as the presence of AV DNA has been identified in breast milk [288]. However, no association was observed between the infant’s breastfeeding status and AV richness [289].

It is noteworthy that a study of interest evidenced the prevalence of TTV in the vaginal ecosystem of pregnant women, thus representing a possible predictor of local immune status. Indeed, the presence and load of the virus vary according to local vaginal conditions, being more prevalent in cases of higher levels of leukocytes, higher levels of microbes related to bacterial vaginosis, and a lack of *Lactobacillus crispatus* [290].

It is noteworthy that some studies have identified alphatorquevirus DNA in blood as early as the second month of life, and also in stool samples in the first months of life, suggesting replication in the gut at a very young age [51]. The peak of AV abundance in the gut was found between the sixth and twelfth months of life, after which the abundance decreased [291]. The same is probably true for the AV virome in the blood (anellome), and the early-life dynamics of the anellome may contribute to the maturation of children’s immune systems. 

## 6. TTV and the Immune System: How Are They Linked?

The interaction between TTV and the immune system represents another challenging area of research, with the mechanisms underlying this close relationship remaining poorly understood. 

Due to the lack of an efficient culture system to support TTV replication, the transcription profile of TTV has been largely gained from human cell lines (COS1, HEK293, and L428) transfected with TTV plasmids [26]. Three spliced mRNAs of TTV that produce at least six proteins by alternative translation initiation have been reported [292]. At present, the functional role of ORF2 protein is well characterized. The overexpression of the TTV ORF2-encoded protein has been shown to suppress NF-κB activation elicited by TNFα in various human cancer cell lines, including HeLa and HepG2, and in the mouse macrophage line RAW 264.7 [15]. Further analyses revealed that the TTV ORF2 protein can suppress NF-κB activity in vitro in a dose-dependent manner, affecting translocation of NF-κB p65 and p50 subunits to the cell nucleus, thus inhibiting the transcription of downstream genes such as interleukin IL-6, IL-8, and cyclooxygenase-2. Together, these findings indicate that the TTV ORF2 protein may be involved in the negative regulation of host cell inflammatory responses. 

There is growing evidence that AVs interact with cells and soluble substances known to support the equilibrium of innate and adaptive immunity, and this interaction can significantly affect an infected host [293,294]. Human AVs, especially TTV, have established a suitable interaction with the host’s immune system, and it has been shown that the replication rate of these viruses can be an appropriate measure to monitor the overall function of the host immune system. Research has also demonstrated the interaction between TTVs and immune molecules known as “pathogen-associated molecular patterns” (PAMP), recognized by pathogen recognition receptors (PRRs), which cause inflammatory and immunological reactions [13,295]. The adaptive immune responses of infected hosts play a critical role in determining the resolution of primary AV infections and the circulation of AVs, such as TTV, in the peripheral blood. As shown, of two chimpanzees inoculated with the same volume of human serum containing TTV, one developed IgM and IgG antibodies to the virus, while the other, which developed no detectable antibodies, became persistently infected [13,294,296,297]. The information that is now available concerning humoral responses suggests that TTV induces antiviral antibodies that, at least in most cases, fail to eradicate the virus. 

Toll-like receptors (TLRs) belong to a group of cell-surface proteins that are crucial for identifying a wide range of pathogens and initiating an innate immune response. TLR9 recognizes intracellular unmethylated heterodimers of guanosine and cytosine (CpGs), which are abundant in the genomes of DNA viruses. Depending on the number of nucleotides flanking CpGs, this may stimulate the production of either pro- or anti-inflammatory cytokines [270]. It has been reported that the genome as well as the replicative intermediates of anellovirus are unusually rich in CpG sequences [295]. The DNA of genogroup 1 of anellovirus (ViPiSAL strain) was found to provoke the robust activation of TLR9 and the production of proinflammatory cytokines in ex vivo mouse spleen cells [270]. Nevertheless, the genomes of other anellovirus strains failed to promote inflammatory responses. These findings may indicate that the effects of anelloviruses on the host’s inflammatory status vary depending on genogroups.

Evidence suggests that TTV encodes microRNAs (miRNA) that cooperate with viral proteins to regulate the expression of viral genes involved in replication, pathogenesis, inflammation, and immune evasion. The functional relevance of proteins translated from other TTV ORFs and TTV-encoded miRNAs warrants further study. 

Another fundamental aspect that remains to be elucidated is the location of TTV persistence. However, its ubiquitous presence suggests that granulocytes could be the reservoir for TTV.

In a study by Kosulin et al. [33], the potential site of TTV replication in different leukocyte subsets was assessed by flow-sorting separation. This study evidenced granulocytes as a site of TTV persistence without any correlation between TTV and T lymphocyte count. Conversely, other studies have utilized molecular methods to demonstrate the presence of TTV in the bloodstream and have shown that the highest levels of TTV are found in patients living with HIV who have a low CD4 T lymphocyte count [298]. Honorato et al. studied the possible correlation between the presence of TTV in saliva and circulating CD4+ T lymphocytes in HIV patients. In this case too, the levels of TTV in saliva had an inverse correlation with CD4+ T lymphocytes, but a direct correlation with HIV viremia [255].

It is evident that these results once again serve to demonstrate the utility of TTV in the monitoring of immunosuppression. However, it must be noted that this utility is predicated on an absence of an understanding of the underlying mechanism.

## 7. TTV Detection: From Traditional to Innovative Screening Methods

TTV detection and quantification have been important for understanding its epidemiology, potential clinical relevance, and role as a marker of immune competence. Various methodologies have been employed for TTV screening over the years, ranging from traditional PCR-based techniques to innovative molecular approaches that enhance sensitivity, specificity, and reproducibility. Despite these advancements, standardization remains a significant challenge affecting the comparability of results across different studies and laboratories.

The most widely used method for TTV detection is quantitative PCR (qPCR), which provides a sensitive and specific approach for measuring viral loads in clinical and research settings. qPCR assays typically target conserved regions of the TTV genome, such as the untranslated region (UTR), which is less affected by the virus’s extensive genetic diversity. Several commercial and in-house qPCR assays have been developed, including those by Maggi et al. [58] and Okamoto et al. [44], which are often used in studies assessing TTV viremia as a potential biomarker for immune function. Among commercial assays, the bioMérieux ARGENE^®^ TTV R-GENE^®^ kit (bioMérieux S.A., Marcy l’Etoile, France)is commonly used in clinical laboratories for standardized TTV quantification. This assay provides reproducible viral load measurements and has been applied in monitoring TTV viremia in transplant recipients to assess immune suppression levels. qPCR is extensively applied in monitoring TTV loads in organ transplant patients, where high viremia has been correlated with immunosuppression levels, offering a non-invasive tool for optimizing therapy [27,106].

Nested PCR was one of the first molecular techniques used to detect TTV, and remains valuable for research applications requiring high sensitivity. This method involves two rounds of PCR amplification, enhancing the ability to detect low viral loads, which is particularly useful for identifying TTV in samples with minimal viral DNA. Early studies on TTV prevalence relied on nested PCR, with Nishizawa et al. [1] using this approach to characterize TTV sequences in human serum. However, nested PCR has significant limitations, including a high risk of cross-contamination due to multiple amplification steps and the lack of quantitative capabilities, making it unsuitable for clinical monitoring.

More recently, digital droplet PCR (ddPCR) has emerged as a tool for the absolute quantification of TTV DNA. Unlike qPCR, which relies on standard curves for quantification, ddPCR partitions the sample into thousands of nanoliter-sized droplets, allowing for a more precise and absolute measurement of viral DNA copies. This technique has been applied in research investigating the role of TTV as an immune monitoring biomarker in organ transplantation, showing improved reproducibility over qPCR. For example, Schmitz et al. [212] demonstrated that ddPCR provided a more accurate quantification of TTV loads in transplant recipients, minimizing variability due to primer mismatches. Additionally, a ddPCR assay developed by Maggi et al. [299] at the University of Pisa has been successfully applied for TTV quantification in different clinical settings, including transplant patients and immunocompromised individuals. Their work demonstrated that ddPCR offers improved sensitivity and reproducibility compared to qPCR, particularly in cases where precise viral load monitoring is required. Despite its advantages, ddPCR remains costly and technically complex, limiting its widespread adoption in routine diagnostics

Although molecular methods dominate TTV diagnostics, serological approaches have been explored to assess immune responses against the virus. However, the identification of reliable TTV antigenic targets has been challenging due to the virus’s genetic variability and unclear interaction with the immune system. Some studies have attempted to develop ELISA-based assays for detecting TTV-specific antibodies, but these have not gained widespread use due to the poor correlation between seropositivity and viral load [300].

Next-generation sequencing (NGS) and metagenomic approaches have also advanced TTV research, enabling the discovery of novel TTV species and comprehensive virome analyses. These techniques have been particularly useful in epidemiological studies exploring the diversity of TTV strains across different populations and environments. For example, the metagenomic sequencing of blood and respiratory samples has revealed the co-presence of multiple TTV species, suggesting a dynamic and complex viral population structure. Furthermore, wastewater-based surveillance using NGS has been employed to monitor TTV circulation at the population level, highlighting its potential as a public health marker. However, these approaches require significant bioinformatics expertise, have high associated costs, and are not yet suitable for routine clinical applications [164].

Another emerging technology for TTV detection is CRISPR-based diagnostics, leveraging the sequence-specific recognition capabilities of CRISPR-Cas systems. CRISPR-Cas12 and Cas13 enzymes can be programmed to detect specific TTV DNA sequences with high specificity and sensitivity. This technology has been explored for the rapid detection of viral infections, with potential applications in point-of-care diagnostics [301]. While CRISPR-based assays for TTV are still in the early stages of development, they hold promise for future clinical use, particularly in settings requiring rapid and accurate viral detection.

A major challenge in TTV screening is the lack of standardization, which affects the comparability of viral load measurements across different laboratories and clinical studies. Variability in primer design, detection platforms, and analytical workflows has led to inconsistent results, making it difficult to establish universal clinical cutoffs for TTV quantification. To address this issue, the European TTXGUIDE project has been launched with the goal of developing harmonized protocols for TTV detection and quantification. This initiative is part of a broader effort to optimize immunosuppressive therapy in transplant patients by using TTV levels as a biomarker of immune function. Within TTXGUIDE, transplant recipients are monitored for TTV viremia, and viral load measurements are used to adjust immunosuppressive treatment to prevent both rejection and opportunistic infections. As part of this project, efforts are being made to establish standardized qPCR assays, reference materials for viral load calibration, and clinically relevant thresholds to ensure reliable and reproducible TTV quantification. By creating a unified framework for TTV-based immune monitoring, TTXGUIDE aims to facilitate the integration of TTV measurements into routine clinical practice, enhancing its reliability as a diagnostic and prognostic tool in transplant medicine.

In conclusion, TTV detection has evolved from early PCR-based methods to sophisticated molecular techniques, each with distinct advantages and limitations. While qPCR remains the gold standard for clinical applications, newer approaches like ddPCR, NGS, and CRISPR-based diagnostics offer promising avenues for improving sensitivity, specificity, and reproducibility. Addressing the challenges of standardization will be essential for enhancing the clinical utility of TTV screening, particularly in areas such as transplant medicine, immune monitoring, and virome research.

## 8. A Step Forward: The Future of TTV

AVs have evolved over millions of years and have developed several distinctive traits that make them ideal candidates for use as viral therapeutics.

As a ubiquitous component of the human virome, AVs are characterized by their lack of pathogenicity. These properties render them optimal candidates for use as vectors in the development of next-generation genetic medicines.

In addition, AVs are among the most genetically diverse and pantropic components, suggesting that they could be used as a gene therapy vector platform with broad tropism for multiple disease sites. Additionally, the extensively documented ability of AVs to persist and replicate in humans without triggering humoral immune responses could help minimize or avoid the problem of immunogenicity [300].

In recent decades, virologists have dedicated their research to understanding the vast and diverse world of human-associated viruses, leading to the discovery of what has been termed ’viral dark matter’. Remarkably, some of these viruses are harmless and can persist in the human body for long periods without causing any adverse effects. This is a defining characteristic of commensalism, a symbiotic relationship in which one organism benefits while the other remains unaffected. We believe that AVs, which are present in virtually every human being and a wide range of tissues, provide an opportunity to create a programmable platform for the development of viral therapeutics capable of safely treating a wide range of diseases with greater precision and dose adjustability.

In addition, TTV does not elicit an immune response, allowing patients to be treated with multiple doses, thus overcoming the major obstacle to viral-based therapies, namely the inability to administer repeated doses due to the generation of a strong immune response to each subsequent exposure [292]. 

Finally, because TTV is a ubiquitous virus, its ability to target inaccessible tissues has the potential to open up solutions for a wide range of diseases by using multiple routes of administration and ensuring the sustained expression of therapeutic proteins, but without the potentially harmful integration into the human genome, as AVs remain as episomes in the nucleus [301].

## Figures and Tables

**Table 1:** Papers published from 1998 to the present regarding (Anellovirus) OR (Torque Teno Virus) associated with various clinical conditions.

			(Anellovirus) or (Torque Teno Virus) as		
			Prognostic Marker	Immune and Prognostic Marker	Causal Factor
Transplant	Liver	23	[13,91,92,93,94,95,96,97,98,99,100,101,102,103,104]	[67,68,105,106,107,108,109,110]	[111,112,113,114,115,116,117,118]
	Kidney	74	[119,120,121]	[8,27,61,63,67,71,72,74,106,122,123,124,125,126,127,128,129,130,131,132,133,134,135,136,137,138,139,140,141,142,143,144,145,146,147,148,149,150,151,152,153,154,155,156,157,158,159,160,161,162]	[13,102,118,163,164,165,166]
	Lung	24	[6,167,168]	[6,62,66,73,77,127,144,169,170,171,172,173,174,175,176,177,178,179,180,181,182]	[183,184]
	Heart	4		[70,172,185,186]	
	Pancreas	1		[105]	
	Bone marrow	4		[98,104,187,188]	
	Hematopoietic stem cell	27	[189,190]	[191,192,193,194,195,196,197,198,199,200,201,202,203,204,205,206,207,208,209,210]	[33,211,212,213,214]
Cancer	Melanoma	2	[215]	[78]	
	Colorectal	2	[216,217]		
	Hepatocellular	25	[83,218,219,220,221]	[222]	[223,224,225,226,227,228,229,230,231,232,233]
	Cervical	6	[234,235,236]	[237,238]	[217]
	Lung	2	-	[89,239]	-
	Pancreatic	1	[84]	-	-
	Head and neck	4	[240,241]	[81]	[242]
	Laryngeal	2	[85]	-	[220]
	Leukemia	4	[243,244,245]	-	[220]
	Lymphoma	14	[19,83,246,247,248,249,250,251,252]	[98,112]	[253]
	Kaposi sarcoma	2	-	[254,255]	-
Immunosenescence		5	-	[256,257,258,259]	-
Allergy		2	[260]	[261]	-
Inflammatory diseases		10	[260]	[80,110,262,263,264,265,266,267,268,269,270,271]	-
Autoimmunity		10	-	[272,273,274,275,276,277,278]	[279,280,281]

## Data Availability

No new data were created or analyzed in this study.
